# Blood cell parameters for screening and diagnosis of hereditary spherocytosis

**DOI:** 10.1002/jcla.22844

**Published:** 2019-04-03

**Authors:** Lin Liao, Yuchan Xu, Hongying Wei, Yuling Qiu, Wenqiang Chen, Jian Huang, Yifeng Tao, Xuelian Deng, Zengfu Deng, Hui Tao, Faquan Lin

**Affiliations:** ^1^ Department of Clinical Laboratory The First Affiliated Hospital of Guangxi Medical University Nanning China; ^2^ Department of Pediatric The First Affiliated Hospital of Guangxi Medical University Nanning China; ^3^ Department of Pediatric Laboratory The First Affiliated Hospital of Guangxi Medical University Nanning China; ^4^ Department of Clinical Laboratory Affiliated Tumor Hospital of Guangxi Medical University Nanning China

**Keywords:** blood cell parameter, hemolytic anemia, hereditary spherocytosis, mean reticulocyte volume, mean sphered corpuscular volume

## Abstract

**Background:**

There is currently no single index for the diagnostic screening of hereditary spherocytosis (HS). However, hematology analyzers are widely used in hospital laboratories because of their highly automated performance and quality control procedure, and detection of some blood cell parameters may be useful for the early screening of HS.

**Methods:**

We investigated the values of blood cell parameters for the screening and differential diagnosis of HS. We performed a descriptive study of 482 samples (67 cases of HS, 59 cases of G6PD deficiency, 57 cases of AIHA, 199 cases of thalassemia, and 100 cases of healthy controls) that were run on Beckman Coulter LH780 Hematology Analyzer.

**Results:**

HS was characterized by increased MCHC, decreased MRV, MSCV‐MCV < 0, and increased Ret with no concomitant increase in IRF. The areas under the ROC curves were MSCV‐MCV (0.97; 95% CI 0.95‐1.0) > MRV (0.94; 95% CI 0.91‐0.97) > MCHC (0.92; 95% CI 0.88‐0.97) > Ret/IRF (0.77; 95% CI 0.7‐0.84). MSCV‐MCV ≤ 0.6 fl was valuable parameter for the diagnostic screening of HS, with a sensitivity of 95.5% and specificity of 94.9%.

**Conclusion:**

These indices have high reference values for differentiating HS from thalassemia, AIHA, and G6PD deficiency.

## 1 INTRODUCTION

Hereditary hemolytic anemia is the most common hematologic disease worldwide, while the reported frequency of glucose‐6‐phosphate dehydrogenase (G6PD) deficiency is about 8% in malaria‐endemic countries, equivalent to approximately 1.33 hundred million women and 2.2 hundred million men.^1^ It is estimated that approximately 7% of the global population are carriers of inherited hemoglobin disorders. About 14 504 newborn infants suffer from hemoglobin H disease annually and 23 329 from severe thalassemia (THAL), most in developing countries.^2^, ^3^ The incidence of hereditary spherocytosis (HS) in North America and northern Europe is 1/2000–1/5000.^4^, ^5^ There are no available population census data regarding HS, but several related studies have been conducted. Li et  al^6^ reported that 112 of 356 (31.5%) patients diagnosed with hereditary hemolytic anemia had HS, while Wang et  al^7^ found that 18% of 140 pediatric patients with hemolytic anemia had HS, a proportion only exceeded by autoimmune hemolytic anemia. Diagnosis of HS depends on family medical history, clinical manifestations, and laboratory examinations. Laboratory tests used to diagnose HS include red cell morphology examination, blood cell parameters, red cell osmotic fragility test, red cell membrane protein measurement, and membrane protein mutation detection. HS is characterized by an increased number of microspherocytes in the peripheral blood, with hyperchromatic small red cells being of particularly high diagnostic value. The presence of microcytic erythrocytes > 7.8% can be used for HS diagnosis with a sensitivity of 56.7% and specificity of 84.8%.^8^ However, red cells shrink and are misshapen in patients with autoimmune hemolytic anemia (AIHA), and different numbers of microspherocytes may be present, thus reducing the specificity of red cell morphology for HS diagnosis. The current laboratory tests used to screen HS include the NaCl osmotic fragility test, acidified glycerol lysis test (AGLT), and sucrose hemolysis test. However, the NaCl osmotic fragility test has low sensitivity of 48%‐95% because of different types of membrane protein deficiencies, making it unsuitable for screening of mild HS.^9^ The sensitivity of the AGLT50 is higher than the NaCl osmotic fragility test; however, although AGLT50 is relatively short in patients with HS with a diagnostic sensitivity of 95%,^10^ it is also shortened in patients with AIHA.^11^ Regarding the sucrose hemolysis test, hemolysis > 2.8% has a diagnostic sensitivity of 78.7% and specificity of 95.3% for HS,^12^ but is a complex procedure. A next‐generation osmotic gradient ektacytometry (NG‐OGE) assay was recently introduced for the screening of HS. The NG‐OGE is useful for distinguishing HS from other hereditary red cell membrane disorders; however, it does not differentiate between HS and AIHA.^13^, ^14^ Unfortunately, the new generation of ektacytometer is difficult to be applied in the clinical laboratory, mainly used in some research centers or specialized laboratories. Red blood cell membrane protein detection tests include the eosin‐5'‐maleimide binding test and sodium dodecyl sulfate‐polyacrylamide gel electrophoresis test. The former has a sensitivity of 93% and specificity of 98% for the diagnosis of HS,^11^ and the latter is more specific for protein 4.2– and ankyrin‐deficient HS, but less specific for asymptomatic and mild HS, and is thus unsuitable for determining the type of membrane protein deficiency in approximately 10% of HS patients.^15^ Gene mutations in HS are scattered, and no specific mutation sites suitable for screening have yet been identified.

Hematology analyzers are widely used in hospital laboratories because of their highly automated performance and quality control procedures, and some blood cell parameters may be suitable for early screening of HS. Mean corpuscular hemoglobin concentration (MCHC) was included as one of the diagnostic criteria for HS in guideline for the diagnosis and management of HS in 2004,^16^ and mean sphered corpuscular volume (MSCV) has also been reported to have diagnostic value in HS.^17^, ^18^, ^19^ Furthermore, we recently found that mean reticulocyte volume (MRV) was a valuable index for the diagnosis of HS.^20^ In this study, we investigated the values of blood cell parameters including MRV, MSCV, MCHC, and absolute reticulocyte count (Ret)/immature reticulocyte fraction (IRF) for the screening and differential diagnosis of HS.

## 2 MATERIALS AND METHODS

### 2.1 Subjects

This study was approved by the Ethics Committees of the First Affiliated Hospital, Guangxi Medical University in China. All subjects gave their informed consent to participate in the study. A total of 67 patients, including 37 men and 30 women (median age 17.0 years， mean age 18.6 ± 15.6 years), were diagnosed with HS from June 2012 to May 2014. HS was diagnosed based on their clinical symptoms, biochemistry, complete blood cell count, peripheral blood smear examination, osmotic fragility (OF) test, and other laboratory examinations. We have conducted family study on the clearly diagnosed patients, so as to further screen out more HS patients. The disease control group comprised patients with THAL, G6PD deficiency anemia, and AIHA. One hundred and ninety‐nine patients with THAL (105 men, 94 women; median age 21.0 years， mean age 23.5 ± 19.8 years) were diagnosed by genetic detection and clinical manifestations. Fifty‐nine patients with G6PD deficiency anemia (47 men, 12 women; median age 28.0 years， mean age 28.8 ± 25.1 years) were diagnosed through G6PD activity detection combined with family history, contact history, and clinical manifestations. Fifty‐seven patients with AIHA (23 men and 34 women; median age 32.0 years， mean age 37.8 ± 22.1 years) were diagnosed by anti‐human globulin test (ie, Coombs test), cold agglutinin test, and clinical manifestations. A normal control group comprising 50 men and 50 women (median age 40.0 years， mean age 37.8 ± 20.6 years) underwent physical examination and was not treated.

### 2.2 Equipment and methods

No therapeutic measures were given to patients in the HS group or the disease control group. Two milliliters of fasting venous blood was taken from each subject and transferred to an anticoagulation tube containing 10% EDTA‐K2 (1.5 mg/mL) for analysis of red blood cell and reticulocyte parameters using a Coulter LH780 Hematology Analyzer (Beckman Coulter Inc, Fullerton, CA, USA). During analysis, methylene‐blue‐stained red blood cells formed deproteinated spherocytes after treatment with an acidic hypoosmotic solution. The spherocytes could then be further divided into mature red blood cells and reticulocytes using the volume, conductivity, and light scatter technology. The mean volume of the spherocytes was measured as MSCV, and MCV, MCHC, MRV, and Ret of red blood cells and reticulocytes were detected simultaneously. The IRF was calculated by measuring the fluorescence intensity of RNA left in the reticulocytes using a Coulter LH780 Hematology Analyzer.

### 2.3 Statistical analysis

All data were analyzed statistically using SPSS 17.0 (SPSS, Chicago, IL, USA) and GraphPad Prism 5.0 (San Diego, CA, USA). Measurement data were expressed as mean ± standard deviation, and means of each parameter were compared between samples by one‐way analysis of variance. Normally distributed data were compared by least significant difference and non‐normally distributed data by Tamhane’s T2 tests. A level of *P* < 0.05 was considered statistically significant. The area under the receiver operating characteristic (ROC) curve was calculated, and the diagnostic values of MCHC, MRV, MSCV‐MCV, and Ret/IRF for the diagnosis of HS were compared. The optimal diagnostic cutoff point for HS and the corresponding sensitivity and specificity were determined by analyzing the sensitivity and misdiagnosis rate of the ROC curve.

## 3 RESULTS

**Figure 1:**
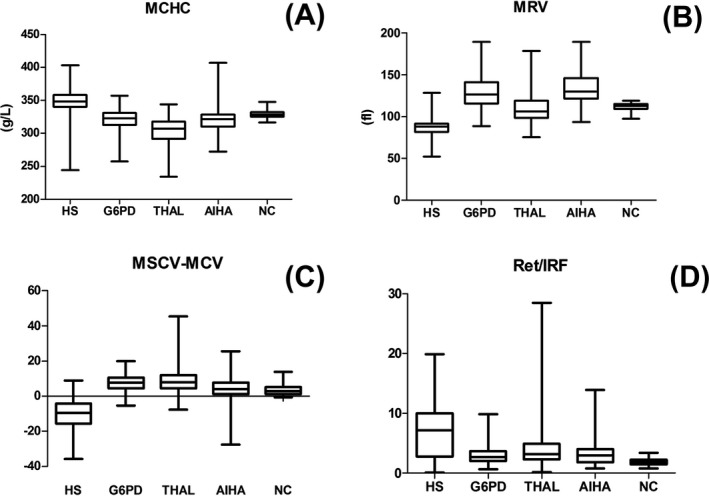
Comparisons of MCHC (A), MRV (B), MSCV‐MCV (C), and Ret/IRF (D) among disease control, normal control, and HS groups. The heavy lines and boxes indicate the median and interquartile range, respectively. Values below the 25th percentile and above the 75th percentile represent 50% of the data. MCHC and Ret/IRF were significantly higher in the HS group compared with the other groups (*P *< 0.01), while MRV and MSCV‐MCV were significantly lower (*P *< 0.01). HS, hereditary spherocytosis; MCHC, mean corpuscular hemoglobin concentration; MRV, mean reticulocyte volume; MSCV‐MCV, mean spherical corpuscular volume‐mean corpuscular volume; NC: normal control; Ret/IRF, absolute reticulocyte count/immature reticulocyte fraction.

Blood cell parameters in the HS, disease control, and normal control groups are shown in Table 1. MCHC and Ret/IRF were significantly greater in the HS group, while MRV and MSCV‐MCV were significantly lower, compared with the disease control and normal control groups (all *P* < 0.01). The distributions of MCHC, Ret/IRF, MRV, and MSCV‐MCV in the HS, disease control, and normal control groups are shown in Figure 1.

**Table 1:** Blood cell parameters in HS, disease control, and normal control groups

Parameter	HS (*n* = 67)	G6PD deficiency anemia (*n *= 59)	Thalassemia (*n *= 199)	Autoimmune hemolytic anemia (*n *= 57)	Normal control (*n *= 100)
Hb (g/L)	98.9 ± 24.9	96.1 ± 23.6	86.5 ± 21.9	70.1 ± 20.2	140.7 ± 13.4
MCHC (g/L)	347.7 ± 21.3	320.4 ± 14.9	303.2 ± 21.7	318.5 ± 17.4	328.4 ± 5.9
MCV (fl)	84.4 ± 7.0	93.1 ± 14.4	72.6 ± 10.5	91.6 ± 11.3	89.6 ± 3.8
MRV (fl)	87.5 ± 11.1	128.4 ± 20.4	109.6 ± 16.3	132.6 ± 19.7	112.3 ± 4.1
MSCV (fl)	73.6 ± 9.2	100.4 ± 15.0	81.6 ± 10.9	96.6 ± 12.8	93.1 ± 3.8
Ret (10^9^/L)	248.6 ± 171.4	120.6 ± 61.4	119.6 ± 88.4	152.2 ± 127.7	51.7 ± 15.7
Ret%	7.9 ± 5.4	3.7 ± 2.2	3.3 ± 2.9	7.2 ± 6.3	1.2 ± 0.4
IRF (%)	35.1 ± 9.8	41.1 ± 14.1	31.5 ± 11.7	45.4 ± 16.8	28.1 ± 5.3
MSCV‐MCV	‐10.8 ± 9.0	7.3 ± 4.9	8.9 ± 7.5	5.0 ± 6.0	3.60 ± 3.1
Ret/IRF	6.9 ± 4.2	3.2 ± 1.9	4.0 ± 3.3	3.4 ± 2.3	1.9 ± 0.6

Hb, haemoglobin; HS, hereditary spherocytosis; MCHC, mean corpuscular hemoglobin concentration; MCV, mean corpuscular volume; MRV: mean reticulocyte volume; MSCV, mean spherical corpuscular volume; IRF, immature reticulocyte fraction; Ret, reticulocyte absolute count; Ret%: reticulocyte count.

**Figure 2:**
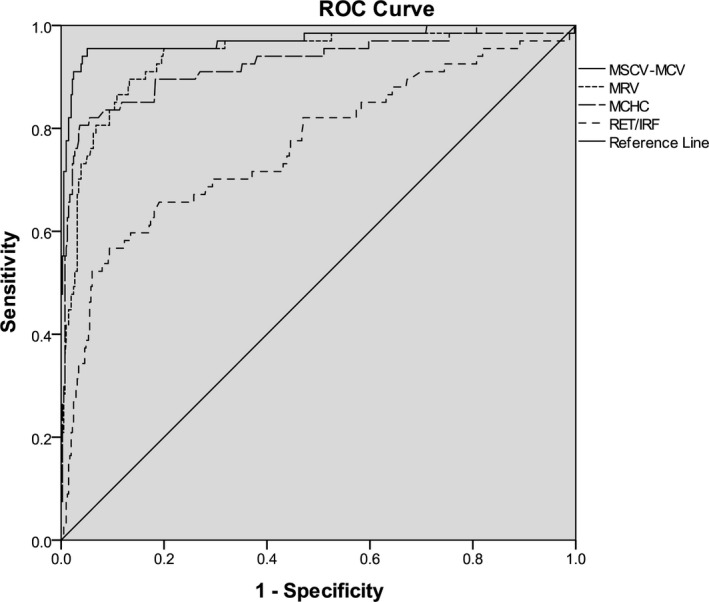
The ROC curve analysis for MSCV‐MCV, MRV, MCHC, and Ret/IRF for HS screening. The area under the ROC curve was greatest for MSCV‐MCV (0.97), followed by MRV (0.94), MCHC (0.92), and Ret/IRF (0.77). HS, hereditary spherocytosis; MCHC, mean corpuscular hemoglobin concentration; MRV, mean reticulocyte volume; MSCV‐MCV, mean spherical corpuscular volume‐mean corpuscular volume; Ret/IRF, absolute reticulocyte count/immature reticulocyte fraction.

The ROC curve analysis for MSCV‐MCV, MRV, MCHC, and Ret/IRF for HS screening is shown in Figure 2 and Table 2. The area under the ROC curve of MSCV‐MCV for diagnosis of HS was 0.97 (95% CI 0.95, 1.00), the sensitivity was 95.5%, and the specificity was 94.9% with a cutoff value of 0.6 fl. The area under the ROC curve of MRV for diagnosis of HS was 0.94 (95% CI 0.91, 0.97), the sensitivity was 86.6%, and the specificity was 89.2% with a cutoff value of 96.1 fl. The area under the ROC curve of MCHC for diagnosis of HS was 0.92 (95% CI 0.88, 0.97), the sensitivity was 82.1%, and the specificity was 94.5% with a cutoff value of 334.9 g/L. The area under the ROC curve of Ret/IRF for diagnosis of HS was 0.77(95% CI 0.70, 0.84), the sensitivity was 65.7%, and the specificity was 81.0% with a cutoff value of 4.5.

**Table 2:** Analysis of ROC curves for blood cell parameters used for HS screening

Parameter	AUC	95% CI		Cutoff point	Sensitivity	Specificity
		Lower limit	Upper limit			
MSCV‐MCV	0.97	0.95	1.00	0.6	95.5%	94.9%
MRV	0.94	0.91	0.97	96.1	86.6%	89.2%
MCHC	0.92	0.88	0.97	334.9	82.1%	94.5%
Ret /IRF	0.77	0.70	0.84	4.5	65.7%	81.0%

AUC, Area under the curve; HS, hereditary spherocytosis; MCHC, mean corpuscular hemoglobin concentration; MRV, mean reticulocyte volume; MSCV‐MCV, mean spherical corpuscular volume‐mean corpuscular volume; Ret/IRF, absolute reticulocyte count/immature reticulocyte fraction.

## 4 DISCUSSION

In patients with HS, red blood cell membrane protein deficiency leads to membrane vesiculation and membrane loss, resulting in a greatly reduced membrane surface area. Because hemoglobin is lost very little compared to the loss of the red blood, and MCHC thus increases with increasing numbers of spherocytes. However, the diagnostic sensitivity and specificity of MCHC are not ideal, with MCHC > 347.0 g/L having a sensitivity of only 73.3% and specificity of 72.6% for the diagnosis of HS.^8^ In addition, an increase in blood mesobilirubin levels interferes with MCHC detection, often leading to false‐positive increases in MCHC levels. Different from the former study, our results showed that MCHC ≥ 334.9g/L had a diagnostic sensitivity of 82.1% and specificity of 94.5%, so it has higher specificity than the last. This may be related to the fact that the control group in this research is mainly the hemolytic anemia, while the increased MCHC is the feature to distinguish HS from other hemolytic anemias, such as THAL and AIHA.

Deproteinized spherocytes are formed after red blood cells are first dyed into new methylene blue and then processed in a kind of acid and hypotonic deproteinized solution. Next, spherocytes are divided into mature erythrocytes and reticulocytes by using VCS technology of Coulter LH780 Hematology Analyzer.^21^ The average volume of all the reticulocytes is MRV, and the average volume of all the red blood cells including mature erythrocytes and reticulocytes is MSCV. MRV is a valuable index for HS screening. Da Costa et  al^22^ compared the membrane surface area of reticulocytes and mature erythrocytes and found that membrane vesiculation and membrane loss occurred at the reticulocyte stage in patients with HS, resulting in greatly reduced membrane surface area and MRV. Lazarova et  al.^23^ examined blood cell parameters including MRV, MSCV, and IRF in 48 patients with HS, seven with AIHA, and 31 with other types of anemia and provided the first evidence for MRV as a valuable screening parameter. MRV ≤ 96.72 fl had a diagnostic sensitivity and specificity of 100% and 88%, respectively. We previously compared blood cell parameters among patients with HS, THAL, AIHA, and G6PD deficiency and identified MRV as a general and specific index for HS screening and for the differential diagnosis of different types of hemolytic anemia.^20^ We also found that MRV ≤ 95.8 fl had a diagnostic sensitivity and specificity of 86.8% and 91.2%, respectively. In the current study, we compared several blood cell parameters for the screening and differential diagnosis of HS and found that the diagnostic value of MRV was second to that of MSCV‐MCV. AIHA and G6PD deficiency are associated with acute hemolysis, leading to compensatory hyperplasia of reticulocytes and promoting the release of immature reticulocytes (prior type IV) and resulting in enlarged MRV. The pathological mechanism underlying THAL involves deletion and mutation of the globin gene, but does not include reduced membrane surface area. THAL thus mainly manifests as chronic hemolysis and anemia, and there was thus no significant difference in MRV between THAL patients and healthy controls.

Red blood cell membrane loss in patients with HS occurs at the reticulocyte stage,^22^ leading to obviously reduced MRV followed by reduced MCV and MSCV, manifested as MSCV‐MCV < 0. In 1999, Chiron et  al^17^ first reported the diagnostic value of MSCV and MCV for HS, with MSCV < MCV showing 100% diagnostic sensitivity and 93.3% specificity for HS. Broséus et  al^18^ used MCV‐MSCV > 9.6 fl as a standard, corresponding to 100% diagnostic sensitivity and 90.57% specificity for HS, while Tao et  al.^24^ reported that MSCV < MCV had a sensitivity of 89.28% and a specificity of 96.14% for the diagnosis of HS. They also pointed out that MSCV < MCV combined with peripheral blood smears, flow cytometry assay, and osmotic fragility testing provided a simple, practical, and accurate screening method for HS. In this study, we compared the diagnostic values of MRV, MSCV‐MCV, MCHC, and Ret/IRF among four disease types and found that MSCV‐MCV was very valuable index for HS screening, with MSCV‐MCV ≤ 0.6 fl having a diagnostic sensitivity of 95.5% and specificity of 94.9%.

Hemolytic anemia is a hyperproliferative anemia, characterized by increased peripheral blood reticulocytes and the appearance of various numbers of nucleated red blood cells. HS is characterized by a high Ret value with no increase in IRF, as reported by Lazarova et  al^23^ However, this characteristic has a lower diagnostic sensitivity and specificity than MSCV‐MCV < 0, MRV, or MCHC.

Some previous studies have investigated the application of multiple blood cell parameters for HS screening, but have used different equipments and blood cell parameters. Lazarova et  al^23^ examined the automated reticulocyte parameters MRV, IRF, and MSCV using a UniCel DxH 800 hematology analyzer (Beckman Coulter, Miami, FL, USA), while Mullier et  al^8^ investigated the value of blood cell parameters MCV, MCHC, Ret, IRF, hypochromic erythrocytes, and microcytic erythrocytes using XE‐2100 and XE‐5000 automated hematology analyzers (Sysmex, Kobe, Japan). They were also the first to consider the three most common types of hemolytic anemia in China (including 56 cases of G6PD deficiency, 109 cases of THAL, and 52 cases of AIHA) as a disease control group and came to a valuable conclusion regarding HS screening and differential diagnosis.

What calls for attention is that there are inevitably some drawbacks and pities in this research. First is the limitation of instrument. MRV can be detected in the hematology analyzers manufactured by multiple companies, including Beckman‐Coulter, Simens and Horiba ABX. Even though, the unification and standardization of the reference range among various instruments remains a problem to be solved. Unfortunately, only Beckman Coulter Hematology Analyzer can detect MSCV, which is limited in the clinical application.^25^, ^26^ Second, the treatment will affect the hemocyte parameters; for instance, HS can be effectively improved after splenectomy, and the anemia in patients with thalassemia can be effectively improved after hematopoietic stem cell transplantation or blood transfusion.^27^ Consequently, the data we adopt in this research are all the examination results of the patients done for a clear diagnosis on admission, and others should take the effect of treatment into consideration when they use our research results for reference.

In conclusion, increased MCHC, decreased MRV, MSCV‐MCV < 0, and increased Ret without increased IRF are characteristic blood cell parameters in HS. Diagnostic guidelines already include MCHC as a criterion for early HS screening, but its diagnostic sensitivity and specificity are not ideal. Nevertheless, it provides a simple and feasible method in hospitals not equipped to perform reticulocyte detection. MSCV‐MCV < 0 and MRV < 96.1 fl are valuable indices with wide application prospects for the differential diagnosis of HS from G6PD deficiency, THAL, and AIHA. Given the widespread use of fully automated hematology analyzers, these blood cell parameters represent potentially valuable tools for the preliminary screening and differential diagnosis of HS.
